# Association of Race/Ethnicity With Emergency Department Destination of Emergency Medical Services Transport

**DOI:** 10.1001/jamanetworkopen.2019.10816

**Published:** 2019-09-06

**Authors:** Amresh D. Hanchate, Michael K. Paasche-Orlow, William E. Baker, Meng-Yun Lin, Souvik Banerjee, James Feldman

**Affiliations:** 1 Section of General Internal Medicine, Boston University School of Medicine, Boston, Massachusetts; 2 Boston Medical Center, Boston, Massachusetts; 3 Department of Emergency Medicine, Boston University School of Medicine, Boston, Massachusetts; 4 Disparities Research Unit, The Mongan Institute, Massachusetts General Hospital, Boston

## Abstract

**Importance:**

Evidence from national studies indicates systematic differences in hospitals in which racial/ethnic minorities receive care, with most care obtained in a small proportion of hospitals. Little is known about the source of these differences.

**Objectives:**

To examine the patterns of emergency department (ED) destination of emergency medical services (EMS) transport according to patient race/ethnicity, and to compare the patterns between those transported by EMS and those who did not use EMS.

**Design, Setting, and Participants:**

This cohort study of US EMS and EDs used Medicare claims data from January 1, 2006, to December 31, 2012. Enrollees aged 66 years or older with continuous fee-for-service Medicare coverage (N = 864 750) were selected for the sample. Zip codes with a sizable count (>10) of Hispanic, non-Hispanic black, and non-Hispanic white enrollees were used for comparison of EMS use across racial/ethnic subgroups. Data on all ED visits, with and without EMS use, were obtained. Data analysis was performed from December 18, 2018, to July 7, 2019.

**Main Outcomes and Measures:**

The main outcome measure was whether an EMS transport destination was the most frequent ED destination among white patients (reference ED). The secondary outcomes were (1) whether the ED destination was a safety-net hospital and (2) the distance of EMS transport from the ED destination.

**Results:**

The study cohort comprised 864 750 Medicare enrollees from 4175 selected zip codes who had 458 701 ED visits using EMS transport. Of these EMS-transported enrollees, 26.1% (127 555) were younger than 75 years, and most were women (302 430 [66.8%]). Overall, the proportion of white patients transported to the reference ED was 61.3% (95% CI, 61.0% to 61.7%); this rate was lower among black enrollees (difference of −5.3%; 95% CI, −6.0% to −4.6%) and Hispanic enrollees (difference of −2.5%; 95% CI, −3.2% to −1.7%). A similar pattern was found among patients with high-risk acute conditions; the proportion transported to the reference ED was 61.5% (95% CI, 60.7% to 62.2%) among white enrollees, whereas this proportion was lower among black enrollees (difference of −6.7%; 95% CI, −8.3% to −5.0%) and Hispanic enrollees (difference of –2.6%; 95% CI, –4.5% to –0.7%). In major US cities, a larger black-white discordance in ED destination was observed (−9.3%; 95% CI, −10.9% to −7.7%). Black and Hispanic patients were more likely to be transported to a safety-net ED compared with their white counterparts; the proportion transported to a safety-net ED among white enrollees (18.5%; 95% CI, 18.1% to 18.7%) was lower compared with that among black enrollees (difference of 2.7%; 95% CI, 2.2% to 3.2%) and Hispanic enrollees (difference of 1.9%; 95% CI, 1.3% to 2.4%). Concordance rates of non-EMS–transported ED visits were statistically significantly lower than for EMS-transported ED visits; the concordance rate among white enrollees of 52.9% (95% CI, 52.1% to 53.6%) was higher compared with that among black enrollees (difference of −4.8%; 95% CI, −6.4% to −3.3%) and Hispanic enrollees (difference of −3.0%; 95% CI, −4.7% to −1.3%).

**Conclusions and Relevance:**

This study found race/ethnicity variation in ED destination for patients using EMS transport, with black and Hispanic patients more likely to be transported to a safety-net hospital ED compared with white patients living in the same zip code.

## Key Points

**Question:**

Are racial/ethnic minorities who use emergency medical services transported to the same emergency department as white residents living in the same zip code?

**Findings:**

In this cohort study of 864 750 Medicare enrollees from 4175 zip codes, the proportion of white patients transported to the reference (or most frequent) emergency department destination was high (61.3%), compared with the proportion of black patients (difference of −5.3%) and Hispanic patients (difference of −2.5%).

**Meaning:**

This study suggests that emergency department destination is substantially different on the basis of the race/ethnicity of patients living in the same zip code.

## Teaser

This cohort study examines the patterns of emergency department destination of emergency medical services transport for black, Hispanic, and white Medicare beneficiaries residing in the same area.

## Introduction

Use of emergency medical services (EMS) aids the rapid evaluation and stabilization of patients and serves the critical function of transportation to a suitable emergency department (ED). Proximity is generally believed to be the primary criteria for identifying the ED destination, but capabilities of the ED are also important for specific conditions (eg, stroke, cardiac events, and trauma). The American College of Emergency Physicians recommends that patients “should be transported to the nearest appropriate ED in accordance with applicable laws, regulations and guidelines.”^1^^(p6)^ However, little population-level evidence is available to date on the extent to which proximity determines the ED destination.^2^

Examining the ED-destination patterns of EMS-transported patients is important because, although the case for rapid transport to an appropriate ED is compelling, other factors may play a role in the ED destination choice. Patients with a history of using inpatient care, especially older adults and those with chronic conditions, may prefer to be transported to their so-called home ED or hospital.^3^ Geography may also be associated with the destination. In areas with multiple EDs in the vicinity, the diffusion of patient transport to different EDs may be greater.

The focus of this study was to measure the differences in ED destination by the race/ethnicity of EMS-transported patients from the same geographic area. We were motivated by the considerable evidence of systematic disparities in the hospitals in which patients in the racial/ethnic minority receive care compared with hospitals frequented by white patients, and these differences are associated with disparities in patient outcomes.^4,5,6,7,8^ These patients include those hospitalized for acute conditions (eg, acute myocardial infarction, congestive heart failure, and pneumonia) for which care is initiated in the ED before admission. Examination of ED destinations of EMS-transported patients provides a potential pathway of differences in hospital destination by race/ethnicity.

Using a national sample cohort of Medicare fee-for-service enrollees, stratified to enable the comparison of patients by race/ethnicity from the same zip code, we examined the ED destination patterns of all of their EMS transports along with the variations in patient severity, chronic illness burden, presence of multiple EDs in the vicinity, and socioeconomic area characteristics. We compared the ED destination patterns among those transported by EMS and those who did not use EMS. We also analyzed the transports to a safety-net ED by race/ethnicity.

## Methods

### Data Sources and Study Cohort

The use of Medicare data for this study was approved by the institutional review board at Boston University. Informed consent was not required because deidentified data were used. We followed the Strengthening the Reporting of Observational Studies in Epidemiology (STROBE) reporting guideline.

From all Medicare enrollees every year from January 1, 2006, to December 31, 2012, we selected those aged 66 years or older with continuous fee-for-service coverage for up to 3 years or until date of death (see eAppendix and eTables 1-4 in the Supplement for details on the identification of the study cohort). To compare EMS use across racial/ethnic subgroups within each zip code, the smallest geographic unit identified in the Medicare data, we followed several steps. First, we stratified all eligible enrollees by their residence zip code (n = 38 423 zip codes) and obtained the count of zip code enrollees by 4 racial/ethnic groups: Hispanic, non-Hispanic black, non-Hispanic white, and other. Second, we identified the subgroup of zip codes with racial/ethnic diversity, defined as containing more than 10 Hispanic, 10 black, and 10 white enrollees each (n = 5606 zip codes). We obtained health care utilization claims data for 2006 to 2012 (up to 3 years of follow-up period) for a stratified random sample of enrollees from the subgroup of zip codes. Third, to ensure an adequate number of EMS transports from each racial/ethnic group from each zip code, we used claims data and identified all EMS transports to any ED in the sample population and included only the zip codes with at least 5 transports for all enrollees of each racial/ethnic group (n = 4175 zip codes); these zip codes contained 34.1% of the overall national eligible enrollee population. The cohort for the present study consisted of a (stratified) random sample of 864 750 enrollees residing in these 4175 zip codes. For secondary analysis, we obtained data on ED visits for the sample population who did not use EMS.

Using American Hospital Association annual survey data for 2006 to 2012, we identified the geographic location of all ED destinations identified in the Medicare claims data and obtained data on the proportion of Medicaid patients served.^9^ We also obtained zip code–level data on population distribution by race/ethnicity and socioeconomic status from the US Census Bureau.^10^

### Outcome Measures

Using data on all EMS transports from each zip code, we identified the most frequent (modal) ED destination among white enrollees as the reference ED destination for the zip code. The main outcome measure was a dichotomous indicator (0 or 1) of whether an EMS transport destination was the reference ED. For the secondary outcomes, we examined (1) whether the ED destination was a safety-net hospital (dichotomous indicator) and (2) the distance (in miles; to convert to kilometers, multiply by 1.6) of EMS transport from the ED destination. To identify safety-net hospitals, we used the Dartmouth Atlas hospital referral regions (n = 306) to find the top quartile of EDs or hospitals in each region used as a safety net in terms of Medicaid share of patients.^11^

### Subgroups and Covariates

As noted in previous studies, the original race/ethnicity field in Medicare data had poor sensitivity in identifying Hispanic patients (33%)^12^; therefore, we used the revised race/ethnicity field, enhanced with surname-based imputation, that offers improved sensitivity (77%).^13,14^ A comprehensive list of surnames that were self-reported as Hispanic by at least 70% of respondents in the US census was applied to Medicare enrollee names to impute additional Hispanic enrollees.^14^ Sensitivity of this race/ethnicity field in identifying non-Hispanic black and white enrollees is more than 97%.

To characterize the differences in acuity of the patient health status, we used the principal diagnosis, with *International Classification of Diseases, Ninth Revision, Clinical Modification* code, reported in the claims for the ED visit after the EMS transport. We identified (1) the dichotomous indicators of 7 high-acuity medical conditions (acute myocardial infarction, congestive heart failure, pneumonia, stroke, sepsis, gastrointestinal bleeding, and arrhythmia); (2) patients with serious injury or trauma, defined as those with an Abbreviated Injury Scale score of 3 or higher (score range: 1 [minor] to 6 [unsurvivable]); and (3) all other admission diagnostic conditions.^15,16^ In addition, we characterized the overall patient morbidity using the 23 condition classification of the Centers for Medicare & Medicaid Services Chronic Condition Data Warehouse,^17,18^ in which the presence of each chronic condition has a distinct dichotomous indicator. We categorized EMS transports according to their Advanced Life Support or Basic Life Support capabilities. Transports with Basic Life Support capabilities include an emergency medical technician, whereas those with Advanced Life Support capabilities also include a paramedic.

We measured socioeconomic status at the patient level, using an indicator of eligibility for Medicaid (ie, dual coverage)^19^ obtained from the claims data, and at the zip code level, using area poverty rate and racial/ethnic minority share (percentage) of area census population obtained from the US Census Bureau’s American Community Survey.^10^

Given that differences in ED destination may be affected by the presence of multiple EDs in the vicinity, we calculated the distance from the centroid of each zip code to each ED to identify the number of EDs within a 10-mile vicinity of each zip code, based on the Euclidean (“as the crow flies”) distance between the zip code centroid and the ED (street) location.^20,21^ As another indicator of advanced care capabilities, EMS transports to EDs of teaching hospitals compared with other EDs were examined using Centers for Medicare & Medicaid Services Cost Reports data.^22^

### Statistical Analysis

The main analytic focus was on estimating the racial/ethnic differences in ED destination of patients from the same zip code transported by EMS. We estimated the regression models of the dichotomous indicator of reference ED destination as the outcome measure; race/ethnicity indicators as the primary covariates of interest; and other covariates to adjust for differences in patient demographics (age, sex), Medicaid eligibility, and year of EMS transport. In addition, to limit comparisons in the outcome measure to patients from the same zip code, all regression models incorporated zip code fixed effects through a distinct dichotomous indicator (0 or 1) for each zip code.^23^ We also incorporated sampling weights to adjust for the stratified sampling by race/ethnicity and zip code.

To accommodate the computational complexity of incorporating the aforementioned specifications, we estimated linear probability regression models of the dichotomous indicator of concordant destination.^23,24,25^ The linear model has the advantage of ease of interpretation in that the model coefficients represent the proportion of each subgroup with concordant ED destination. We obtained heteroskedasticity-consistent robust SE estimates and assessed statistical significance at a 2-sided *P* < .05 level.^23^ Applying this model, we estimated differences by race/ethnicity in rates of reference ED destination for all EMS transports and for subgroups of transports stratified by patient demographics, acuity of medical condition, serious injury or trauma, Advanced Life Support or Basic Life Support capability, area poverty, area minority population share, number of EDs in zip code vicinity, and teaching vs nonteaching hospital ED destination.

We estimated similar linear models for the secondary outcomes: distance of EMS transport, reference ED destination for visits not using EMS, and proportion transported to the largest safety-net ED. All estimation was performed with Stata, version 14.1 (StataCorp LLC).^26^ Data analysis was performed from December 18, 2018, to July 7, 2019.

## Results

The study cohort of 864 750 Medicare enrollees from 4175 zip codes used 458 701 EMS transports to EDs from January 1, 2006, through December 31, 2012 (Table 1). Of these EMS-transported enrollees, 26.1% (127 555) were younger than 75 years, and most were women (302 430 [66.8%]). Black and Hispanic enrollees were younger than their white counterparts. The proportion of patients younger than 75 years was 37.4% (55 811) for black enrollees, 36.4% (32 815) for Hispanic enrollees, and 23.5% (55 813) for white enrollees. The proportion of transports for patients with high-acuity conditions, as identified either by the resulting inpatient admission or by ED principal diagnosis, was largely similar across race/ethnic groups.

**Table 1.** Characteristics of Emergency Medical Services–Transported Emergency Department Visits, 2006-2012^a^^,^^b^

Variable	Patients, No. (%)			
	All	Non-Hispanic		Hispanic
		White	Black	
No. of ED visits	458 701	219 662	137 900	87 053
Advanced life support EMS transports	270 987 (64.8)	123 899 (65.6)	84 722 (61.0)	54 286 (61.74)
Age group, y				
66-74	149 178 (26.1)	55 813 (23.5)	55 811 (37.4)	32 815 (36.4)
75-84	181 968 (41.3)	88 087 (41.6)	52 402 (38.3)	35 402 (41.3)
≥85	127 555 (32.6)	75 762 (34.9)	29 483 (23.8)	29 483 (22.3)
Female sex	302 430 (66.8)	146 699 (67.0)	90 379 (67.0)	56 426 (65.5)
Medicaid (dual coverage) eligible	196 054 (26.7)	60 254 (18.9)	74 760 (51.8)	52 559 (66.8)
ED visit resulting in hospital admission	256 349 (55.1)	127 247 (55.1)	75 202 (55.0)	46 028 (54.3)
ED diagnosis				
Acute myocardial infarction	7142 (1.7)	3731 (1.8)	1679 (1.1)	1456 (1.8)
Congestive heart failure	18 447 (3.7)	8680 (3.6)	5986 (4.4)	3284 (3.7)
Pneumonia	14 116 (3.1)	7592 (3.2)	3482 (2.3)	2566 (3.1)
Stroke	8952 (2.1)	3977 (2.1)	2952 (2.3)	1705 (2.0)
Sepsis	12 470 (2.9)	5240 (2.8)	4217 (3.2)	2624 (3.3)
Gastrointestinal bleeding	8562 (1.8)	3837 (1.8)	2987 (2.2)	1481 (1.8)
Arrhythmia	15 072 (3.8)	8375 (4.0)	1906 (2.8)	2482 (2.9)
Serious injury or trauma	13 445 (4.0)	8542 (4.5)	1906 (1.4)	2482 (3.0)
Other	360 495 (77.0)	169 688 (76.4)	110 855 (80.4)	69 021 (78.5)
Area characteristics				
No. of EDs in 10-mile vicinity				
0-1	89 089 (28.2)	35 085 (30.4)	30 237 (18.8)	20 921 (20.3)
2-4	163 287 (37.5)	80 599 (39.4)	46 551 (31.2)	32 000 (29.4)
≥5	206 325 (34.3)	103 978 (30.2)	61 112 (50.0)	34 132 (50.3)
Destination is teaching hospital ED	277 100 (50.0)	137 564 (47.4)	81 667 (63.0)	49 007 (56.1)
Urban location				
Zip code in largest 16 cities	92 120 (13.4)	40 909 (10.4)	31 565 (23.0)	15 623 (28.5)
Other zip codes	366 581 (86.6)	178 753 (89.6)	106 335 (77.0)	71 430 (71.5)
Zip codes of households in poverty				
Lowest poverty tertile	142 192 (34.6)	73 581 (37.9)	37 955 (18.0)	25 959 (24.5)
Second tertile	125 636 (32.8)	57 832 (33.9)	38 907 (26.5)	25 457 (31.0)
Highest poverty tertile	190 788 (32.6)	88 232 (28.2)	61 013 (55.5)	35 614 (44.5)
Zip codes with >25% census population of				
Black	88 379 (16.3)	32 840 (10.8)	34 878 (56.0)	18 027 (15.0)
Hispanic	139 185 (22.7)	57 294 (18.2)	47 366 (27.0)	30 899 (67.3)

Abbreviations: ED, emergency department; EMS, emergency medical services.

SI conversion factor: To convert miles to kilometers, multiply by 1.6.

^a^ The tabulated percentage estimates were adjusted for stratified sampling.

^b^ The 16 largest US cities were Austin, Texas; Boston, Massachusetts; Chicago, Illinois; Columbus, Ohio; Dallas, Texas; Houston, Texas; Indianapolis, Indiana, Jacksonville, Florida; Los Angeles, California; New York, New York; Philadelphia, Pennsylvania; Phoenix, Arizona; San Antonio, Texas; San Diego, California; San Francisco, California; and San Jose, California. Only the zip codes within the city (not metropolitan area) were included.

Prevalence of chronic conditions varied by race/ethnicity, with higher rates among black enrollees of hypertension (94.7% [129 706]), diabetes (61.6% [83 671]), and kidney disease (51.8% [71 362]) and higher rates among white enrollees of hyperlipidemia (62.1% [132 344]), ischemic heart disease (67.1% [91 512]), and atrial fibrillation (25.7% [57 286]) (eTable 5 in the Supplement).

A larger proportion of the transports for black and Hispanic enrollees compared with those for white enrollees were from the 16 largest US cities (31 565 of 137 900 black enrollees [23.0%] and 15 623 of 87 053 Hispanic enrollees [28.5%], vs 40 909 of 219 662 white enrollees [10.4%]) and from zip codes with 5 or more EDs in a 10-mile vicinity (61 112 black enrollees [50.0%] and 34 132 Hispanic enrollees [50.3%] vs 103 978 white enrollees [30.2%]). (The 16 largest US cities are Austin, Texas; Boston, Massachusetts; Chicago, Illinois; Columbus, Ohio; Dallas, Texas; Houston, Texas; Indianapolis, Indiana, Jacksonville, Florida; Los Angeles, California; New York, New York; Philadelphia, Pennsylvania; Phoenix, Arizona; San Antonio, Texas; San Diego, California; San Francisco, California; and San Jose, California.)

More black and Hispanic enrollees had poorer socioeconomic status, as measured by Medicaid dual-coverage eligibility (74 760 black enrollees [51.8%] and 55 559 Hispanic enrollees [66.8%] vs 60 254 white enrollees [18.9%]) and zip code–level poverty rate (61 013 black enrollees [55.5%] and 35 614 Hispanic enrollees [44.5%] were in the highest tertile of zip codes by median household poverty compared with 88 232 white enrollees [28.2%]). Zip codes in which more than 25% of the census population consisted of black individuals accounted for 34 878 EMS transports (56.0%) of black enrollees; similarly, zip codes with more than 25% Hispanic people had 30 899 transports (67.3%) of Hispanic enrollees.

Overall, the proportion of all EMS transports to the most frequent ED destination was 61.3% (95% CI, 61.0% to 61.7%) among white enrollees (reference EDs); the corresponding proportion was 5.3% lower (95% CI, −6.0% to −4.6%) for black enrollees and 2.5% lower (95% CI, −3.2% to −1.7%) among Hispanic enrollees (Table 2; eTable 6 in the Supplement). In major US cities, a larger black-white discordance in ED destination was observed (−9.3%; 95% CI, −10.9% to −7.7%).

**Table 2.** Concordance in and Transport Distance to Emergency Department Destination by Race/Ethnicity^a^

Patient Subgroup	% (95% CI)		
	Non-Hispanic		Hispanic Patients, Estimate of Difference Between Hispanic and White Patients
	White Patients, Estimate of Mean Concordance Rate/Transport Distance	Black Patients, Estimate of Difference Between Black and White Patients	
Concordance rate, % transported to the most frequent ED destination among white patients			
All zip codes	61.3 (61.0 to 61.7)	−5.3 (−6.0 to −4.6)	−2.5 (−3.2 to −1.7)
Subgroup of zip codes by No. of hospital EDs in 10-mile vicinity			
0-1	72.9 (72.3 to 73.6)	−0.6 (−1.7 to 0.6)	−2.6 (−4.0 to −1.2)
2-4	62.4 (61.8 to 63.0)	−5.2 (−6.4 to −4.0)	−2.8 (−4.1 to −1.6)
≥5	51.0 (50.4 to 51.6)	−8.4 (−9.6 to −7.3)	−2.8 (−7.2 to −1.5)
Zip code in 16 largest US cities	46.1 (45.0 to 47.1)	−9.3 (−10.9 to −7.7)	−2.7 (−4.5 to −0.8)
EMS transport distance to ED			
All zip codes	6.53 (5.08 to 7.98)	−0.63 (−1.44 to 0.18)	−0.49 (−1.35 to 0.38)
Subgroup of zip codes by No. of hospital EDs in 10-mile vicinity			
0-1	7.20 (7.10 to 7.30)	−0.40 (−0.60 to −0.19)	−0.29 (−0.57 to −0.02)
2-4	7.90 (4.04 to 11.8)	−1.39 (−3.75 to 0.96)	−0.88 (−3.32 to 1.57)
≥5	4.47 (4.41 to 4.51)	−0.18 (−0.29 to −0.07)	−0.17 (−0.28 to −0.07)
Zip code in 16 largest US cities	4.28 (4.20 to 4.37)	−0.16 (−0.29 to −0.04)	−0.19 (−0.34 to −0.05)

Abbreviations: ED, emergency department; EMS, emergency medical services.

SI conversion factor: To convert miles to kilometers, multiply by 1.6.

^a^ All estimates were obtained from 1 regression of the outcome measure (indicator of concordant destination ED or distance of EMS transport to ED) on race/ethnicity, age, sex, principal diagnosis, comorbidities, year of EMS transport, and zip code. All regressions applied sampling stratification and weights.

Concordance rates varied inversely with the number of EDs in the zip code vicinity. In zip codes with no more than 1 ED in a 10-mile vicinity, the concordance rate among white enrollees was 72.9% (95% CI, 72.3% to 73.6%); this rate was similar among black enrollees and 2.6% lower (95% CI, (−4.0% to −1.2%) among Hispanic enrollees. In zip codes with 5 or more EDs in a 10-mile vicinity, the concordance rate among white enrollees was 51.0% (95% CI, 50.4% to 51.6%); among black enrollees, the concordance rate was 8.4% lower (95% CI, −9.6% to −7.3%) and among Hispanic patients was 2.8% lower (95% CI, −7.2% to −1.5%) than that among white enrollees. A similar pattern in concordance was found in zip codes in the 16 largest US cities. The concordance rate among whites was 46.1% (95% CI, 45.0% to 47.1%), and this rate was 9.3% lower (36.8%; 95% CI, 35.5% to 38.0%) among black patients and 2.7% lower (43.4%; 95% CI, 41.9% to 44.9%) among Hispanic patients.

The mean EMS transport distance to the ED destination, regardless of whether it was the reference ED, was 6.53 miles (95% CI, 5.08 to 7.98) for white enrollees; that distance was similar for black enrollees (difference of −0.63 miles; 95% CI, −1.44 to 0.18) and Hispanic enrollees (difference of −0.49 miles; 95% CI, −1.35 to 0.38). The mean distance was shorter for white patients in the largest cities (4.28 miles; 95% CI, 4.20 to 4.37), with statistically significantly shorter distance (by approximately 0.2 miles) for black patients (difference of −0.16 miles; 95% CI, −0.29 to −0.04) and Hispanic patients (difference of −0.19 miles; 95% CI, −0.34 to −0.05). We found the reference EDs were also the nearest EDs, according to our comparison of the mean (actual) mileage transported for all patients from each zip code (eTable 7 and eFigure in the Supplement). Across all zip codes, the mean (SD) mileage to the reference ED was 4.8 (4.2) miles and to the second most frequent ED destination was 6.03 miles (95% CI, 4.65 to 7.40) farther. Even in the largest cities, the mean (SD) mileage to the reference ED (3.3 [3.2] miles) was shorter by 0.89 mile compared with the mileage to the second most frequent ED destination.

Concordance rates and their differences by race/ethnicity showed similar patterns across patient subgroups by acuity of ED visit, age, sex, socioeconomic status, and area of high minority status (Table 3; eTable 8 in the Supplement). Among white patients, the concordance rates for ED visits were 61.5% (95% CI, 60.7% to 62.2%) for high-risk conditions, 58.3% (95% CI, 57.1% to 59.4%) for serious injury or trauma, and 61.3% (95% CI, 60.9% to 61.7%) for other conditions. For all categories of conditions, the concordance rates for black (difference of −6.7%; 95% CI, −8.3% to −5.0%) and Hispanic (difference of −2.6%; 95% CI, −4.5% to −0.7%) enrollees were lower by similar rates, although the difference among Hispanics for serious injury or trauma did not attain statistical significance. A similar pattern was found for ED visits grouped by patient hospitalization, transport by EMS with Advanced Life Support or Basic Life Support capabilities, transport to a teaching hospital ED, and area-level poverty rate and minority share of area population.

**Table 3.** Concordance in Emergency Department Destination Between White and Nonwhite Patients, by Patient Acuity and Area Factors^a^^,^^b^

Patient Subgroup	% (95% CI)		
	Non-Hispanic		Hispanic Patients, % Difference in Concordance Rate Between Hispanic and White Patients
	White Patients, Estimate	Black Patients, % Difference in Concordance Rate Between Black and White Patients	
By admission condition			
High risk	61.5 (60.7 to 62.2)	−6.7 (−8.3 to −5.0)	−2.6 (−4.5 to −0.7)
Serious injury or trauma	58.3 (57.1 to 59.4)	−8.9 (−14.6 to −3.3)	−1.2 (−3.8 to 6.3)
Other conditions	61.3 (60.9 to 61.7)	−5.0 (−5.7 to −4.2)	−2.3 (−3.1 to −1.5)
By ED discharge status			
Hospitalization	60.5 (60.1 to 61.0)	−6.3 (−7.2 to −5.3)	−3.0 (−4.0 to −2.0)
Outpatient discharge	62.3 (61.8 to 62.9)	−4.3 (−5.3 to −3.3)	−1.8 (−2.9 to −0.7)
By zip code poverty level, %			
Lowest poverty tertile	62.1 (61.4 to 62.7)	−4.3 (−5.6 to −3.0)	−1.5 (−3.0 to −0.1)
Middle poverty tertile	62.7 (61.0 to 63.3)	−4.3 (−5.6 to −3.2)	−2.0 (−3.3 to −0.6)
Highest poverty tertile	59.4 (58.8 to 60.0)	−6.9 (−7.9 to −5.8)	−3.8 (−5.0 to −2.6)
By high-minority areas			
Zip codes with >25% share of census population of non-Hispanic black race/ethnicity	55.3 (54.4 to 56.2)	−5.5 (−6.8 to −4.1)	−1.7 (−3.2 to −0.1)
Zip codes with >25% share of census population of Hispanic ethnicity	58.6 (58.0 to 59.2)	−4.7 (−5.7 to −3.7)	−1.6 (−2.8 to −0.4)

Abbreviations: ED, emergency department.

^a^ High-risk conditions were ED visits with the principal diagnosis of acute myocardial infarction, congestive heart failure, pneumonia, stroke, sepsis, and gastrointestinal bleeding.

^b^ All estimates were obtained from 1 regression of the outcome measure (indicator of concordant destination ED) on race/ethnicity, age, sex, principal diagnosis, comorbidities, year of emergency medical services transport, and zip code. All regressions applied sampling stratification and weights.

For comparison, we estimated the concordance rates of all non-EMS-transported (walk-in) ED visits (n = 989 022) for the study cohort from 2006 to 2012 (Table 4). For white enrollees, the concordance rate for non-EMS-transported ED visits (52.9%; 95% CI, 52.1% to 53.6%) was smaller compared with the EMS-transported ED visits (61.3%; 95% CI, 61.0% to 61.7%). A similar difference was found for ED visit subgroups in the largest cities and by number of EDs in zip code vicinity. Non-EMS-transported ED visit concordance rates for black enrollees (difference of −4.8%; 95% CI, −6.4% to −3.3%) and Hispanic enrollees (difference of −3.0%; 95% CI, −4.7% to −1.3%) were lower compared with the rate for white enrollees.

**Table 4.** Concordance in Emergency Department Destination Among Patients Transported by Non–Emergency Medical Services, by Race/Ethnicity, 2006-2012^a^^,^^b^

Patient Subgroup	% (95% CI)		
	Non-Hispanic		Hispanic Patients, Estimate of Difference Between Hispanic and White Patients (95% CI)
	White Patients, Estimate of Mean Concordance Rate/Transport Distance	Black Patients, Estimate of Difference Between Black and White Patients (95% CI)	
All zip codes	52.9 (52.1 to 53.6)	−4.8 (−6.4 to −3.3)	−3.0 (−4.7 to −1.3)
Subgroup of zip codes by No. of hospital EDs in 10-mile vicinity			
0-1	65.1 (63.8 to 66.3)	−2.6 (−5.4 to 0.3)	−3.1 (−6.6 to 0.3)
2-4	53.4 (52.1 to 54.8)	−3.3 (−6.2 to −0.5)	−2.7 (−5.7 to −0.2)
≥5	44.2 (42.9 to 45.4)	−7.3 (−9.6 to −5.0)	−3.4 (−6.0 to −0.8)
Zip code in 16 largest US cities	43.9 (41.8 to 45.9)	−6.1 (−9.4 to −2.8)	−0.8 (−4.5 to 2.8)

Abbreviation: ED, emergency department.

SI conversion factor: To convert miles to kilometers, multiply by 1.6.

^a^ Although the findings here were for walk-in ED visits, identification of reference ED in each zip code was based on emergency medical services–transported ED visits (as in Table 2 and Table 3), for comparability of concordance rates between emergency medical services–transported and walk-in ED visits.

^b^ Reported concordance rate and differences by race/ethnicity were adjusted for age, sex, principal diagnosis, comorbidities, Medicaid coverage, year of emergency medical services transport, and zip code location.

With regard to EMS transport to a safety-net ED as a secondary outcome, although 18.5% (95% CI, 18.1% to 18.7%) of white enrollees were transported to a safety-net ED, this proportion was higher among black patients (difference of 2.7%; 95% CI, 2.2% to 3.2%) and Hispanic patients (difference of 1.9%; 95% CI, 1.3% to 2.4%) (Table 5). A similar trend was found in the subgroup of zip codes with 5 or more EDs in the 10-mile vicinity (21.0% [95% CI, 20.6% to 21.5%] of white enrollees transported to a safety-net ED vs higher rates among black enrollees [difference of 4.4%; 95% CI, 3.5% to 5.3%] and Hispanic enrollees [difference of 3.5%; 95% CI, 2.5% to 4.5%]). Similar rates and differences were found in zip codes in the largest 16 US cities.

**Table 5.** Rate of Emergency Medical Services Transport to Safety-Net ED^a^

Patient Subgroup	% (95% CI)		
	Non-Hispanic		Hispanic Patients, Estimate of Difference Between Hispanic and White Patients
	White Patients, Adjusted Rate of Transport to Safety-Net ED/Hospital	Black Patients, Estimate of Difference Between Black and White Patients	
All zip codes	18.4 (18.2 to 18.7)	2.7 (2.2 to 3.2)	1.9 (1.3 to 2.4)
Subgroup of zip codes by No. of hospital EDs in 10-mile vicinity			
0-1	15.0 (14.6 to 15.4)	0.3 (−0.5 to 1.1)	−0.1 (−0.9 to 0.8)
2-4	18.5 (18.1 to 18.9)	2.6 (1.7 to 3.4)	1.2 (2.8 to 2.0)
≥5	21.0 (20.6 to 21.5)	4.4 (3.5 to 5.3)	3.5 (2.5 to 4.5)
Urban location by zip code in 16 largest US cities	21.3 (20.6 to 22.1)	4.8 (3.4 to 6.1)	4.1 (2.6 to 5.6)
Zip codes with >25 census population of			
Black	23.2 (22.9 to 23.7)	3.2 (2.6 to 3.8)	2.2 (1.5 to 2.9)
Hispanic	23.5 (23.2 to 23.8)	2.8 (2.3 to 3.2)	2.9 (2.4 to 3.4)

Abbreviation: ED, emergency department.

SI conversion factor: To convert miles to kilometers, multiply by 1.6.

^a^ Reported concordance rate and differences by race/ethnicity were adjusted for age, sex, primary diagnosis, comorbidities, Medicaid coverage, year of emergency medical services transport, and zip code location.

## Discussion

By comparing the ED destination of EMS transports by race/ethnicity using a national sample of Medicare enrollees, this study highlights 2 findings. First, black and Hispanic enrollees were less likely to be transported to the most frequent ED destination for white patients residing in the same zip code (reference ED). Among all racial/ethnic groups, the proportion of patients transported to reference EDs decreased according to the number of EDs in close proximity; however, the divergence differed systematically by race/ethnicity. Second, black and Hispanic enrollees were more likely to be transported to a safety-net ED compared with their white counterparts from the same zip code.

To our knowledge, no previous study has examined ED destination patterns of EMS transports at the population level. The findings indicate that ED or hospital destination of EMS transport may not be guided primarily by proximity, even for patients with highly acute conditions (albeit identified using administrative data). Even in areas with 1 or 0 EDs in a 10-mile vicinity, 72.9% of the patients were transported to the reference ED, implying that more than 27% were transported to other EDs; this proportion increased by the number of EDs in the vicinity. Across all study zip codes, those not transported to the reference ED traveled 1.67 miles farther, compared with those transported to the reference ED; even in the largest cities, this difference amounted to an additional 0.89 mile of travel.

Little previous evidence exists on the divergence in EMS transport destination, and the available evidence is largely limited to transport of patients with trauma.^3,27,28^ These studies found a sizable variation in the implementation of field-triage practices, with seriously injured patients frequently transported to non–trauma hospitals. Analyzing EMS practitioner–reported reasons for the destination choice, a study of EMS transport from the western United States found that patient or family choice was the most common reason (51%), followed by closest facility (21%) and specialty resource center (15%).^3^ Although Medicare administrative data do not include similar information on reasons for the ED destination choice, our findings suggest a substantial role of patient or family choice. Specifically, we found considerable overlap in the ED destination patterns of EMS transports compared with walk-ins. Overall, the proportion seeking care at the reference ED was 61.3% for EMS-transported patients and 52.9% for walk-in patients.

Considerable evidence of the differences in hospitalization patterns by race/ethnicity, for both acute and elective care, was demonstrated.^7,29,30^ The study findings suggest that 1 pathway may be the ED destination of patients transported by EMS. Even when transported from the same geographic area, we found consistent differences by race/ethnicity in ED destinations. In urban areas with multiple EDs in the vicinity, the divergence was larger. In the largest cities, only 36.8% of EMS transports for black enrollees and 43.4% for Hispanic enrollees were to the most common destination for white enrollees. Because the divergence patterns were largely similar between the EMS-transported patients and walk-in patients, one implication is that patient or family choice may be an important determinant of ED destination even among EMS patients. Further research is needed to understand this divergence and its implications for patient outcomes, which could inform the development of better EMS protocols.

A plausible rationale for the role of patient choice is prior outpatient and inpatient care received at the destination ED or hospital; in particular, among patients with high comorbidities, ED destinations may be their home hospitals.^3^ Patient choice based on history of previous utilization may also account for the higher frequency of transportation to safety-net ED among racial/ethnic minority patients. Nevertheless, the role of other factors, including access barriers, should be examined in future research.

We found evidence that the destination ED is not the nearest ED among patients of all racial/ethnic groups, but the implication for patient outcomes is unclear because several considerations are involved. Evaluation of outcomes requires a better understanding of the factors underlying EMS transport destination. To the extent that the destination choice is motivated by patient preference based on prior health care use, continuity of care may offer advantages. Even though all patients have emergent need for care, the advantages of proximity and continuity may vary by the nature and acuity of the medical condition. Further research is needed into the relative role of proximity by patient condition.

### Limitations

This study has several limitations. First, based on the Medicare claims data, we used the patient residence zip code as the location of the start of EMS transport; although not all transports began at home, we expected the discordance frequency (proportion not from the residence) to be relatively small. A national study reported that the frequency of discordance between actual EMS pick-up location and patient residence was 20.2% for those aged 65 to 84 years and 14.5% for those 85 years or older.^31^ Discordance was lower for medical concerns and higher for vehicle crashes, mass casualty events, and traumatic injuries. Furthermore, given that discordance frequency is likely to be similar among racial/ethnic groups residing in the same area, we did not expect discordance to systematically affect the main findings on racial/ethnic differences in ED destination.

Second, we used zip code as the geographic unit as it is the smallest unit in the Medicare claims data. To the extent that systematic racial/ethnic variation exists in residential location within zip codes, ED destination may be different. However, the number of zip codes in the United States (>40 000) far exceeds the number of EDs (approximately 4600)^32^; therefore, in most zip codes, especially those with no ED, the nearest ED is the same ED that serves all residents in that zip code. In addition, we found that racial/ethnic minorities were more likely to be transported to a safety-net ED, which often involved bypassing several zip codes (eFigure in the Supplement).

Third, for certain clinical conditions (eg, trauma, stroke, and cardiac events), guidelines may necessitate transportation to suitable ED destinations, which may not be near the patient residence. Further study using clinically richer data will be helpful in ascertaining whether incidence of such events is higher among black and Hispanic patients compared with white patients and may explain the patterns observed in the present study.

Fourth, the study sample composition was limited to Medicare enrollees residing in zip codes with racial and ethnic diversity. This composition was by design given our intent to make comparisons among racial/ethnic minority enrollees residing in the same zip codes. Nevertheless, the study cohort represented 34.1% of all Medicare enrollees aged 66 years or older.

## Conclusions

Analyzing population representative data, this study found that Medicare enrollees from the same location who used EMS for emergent conditions were transported to different EDs, with increased divergence in areas with multiple EDs in the vicinity. In all geographic areas, a sizable proportion of black and Hispanic patients were transported to different EDs compared with their white counterparts living in the same zip code. Considerable similarity was observed in the pattern of ED destinations between patients transported by EMS and walk-in patients, suggesting that patient choice is a potential determinant. Future research is needed to understand the reasons for the divergence in ED destination by EMS transport and the extent to which this divergence may be associated with patient outcomes; the results of such research may inform the development of better EMS protocols.

## Notes

Supplement. **eAppendix.** Differences in Emergency Department Destination of Emergency Medical Services Transport of Co-located Patients by Race/Ethnicity and Geography **eTable 1.** Racial/Ethnic Composition of Eligible Medicare Enrollees **eTable 2.** Composition of Eligible Enrollees From Zip Codes With Racial/Ethnic Diversity, 2009 **eTable 3.** Sample Size by Year and Follow-up Cohort Composition (Zip Codes With Diversity) **eTable 4.** Sampling Design **eTable 5.** Prevalence of Chronic Conditions at Baseline Study Cohort From Zip Codes With Racial/Ethnic Diversity **eTable 6.** Concordance Rate and EMS Transport Distance to ED by Race/Ethnicity **eTable 7.** Comparison of Average Distance Between First and Second Most Frequent EMS Destination Among non-Hispanic White Patients **eTable 8.** Concordance in Destination ED Between Whites and non-Whites, by Age, Sex and Medicaid Eligibility **eFigure.** Reference Emergency Departments in Boston City Zip Codes **eReferences.**
