# Supplementary material for: Association of Race/Ethnicity With Emergency Department Destination of Emergency Medical Services Transport
Source: JAMA Netw Open. 2019 Sep 6;2(9):e1910816. doi: 10.1001/jamanetworkopen.2019.10816 (PMC6735492; doi:10.1001/jamanetworkopen.2019.10816)
Supplement: Supplement. — eAppendix. Differences in Emergency Department Destination of Emergency Medical Services Transport of Co-located Patients by Race/Ethnicity and Geography eTable 1. Racial/Ethnic Composition of Eligible Medicare Enrollees eTable 2. Composition of Eligible Enrollees From Zip Codes With Racial/Ethnic Diversity, 2009 eTable 3. Sample Size by Year and Follow-up Cohort Composition (Zip Codes With Diversity) eTable 4. Sampling Design eTable 5. Prevalence of Chronic Conditions at Baseline Study Cohort From Zip Codes With Racial/Ethnic Diversity eTable 6. Concordance Rate and EMS Transport Distance to ED by Race/Ethnicity eTable 7. Comparison of Average Distance Between First and Second Most Frequent EMS Destination Among non-Hispanic White Patients eTable 8. Concordance in Destination ED Between Whites and non-Whites, by Age, Sex and Medicaid Eligibility eFigure. Reference Emergency Departments in Boston City Zip Codes eReferences. [file jamanetwopen-2-e1910816-s001.pdf]

Supplementary Online Content

Hanchate AD, Paasche-Orlow MK, Baker WE, Lin M-Y, Banerjee S, Feldman J.

Association of race/ethnicity with emergency department destination of emergency medical services transport. *JAMA Netw Open*. 2019;2(9):e1910816.

doi:10.1001/jamanetworkopen.2019.10816

eAppendix. Differences in Emergency Department Destination of Emergency Medical Services Transport of Co-located Patients by Race/Ethnicity and Geography
eTable 1. Racial/Ethnic Composition of Eligible Medicare Enrollees

eTable 2. Composition of Eligible Enrollees from Zip Codes With Racial/Ethnic Diversity, 2009

eTable 3. Sample Size by Year and Follow-up Cohort Composition (Zip Codes With Diversity)
eTable 4. Sampling Design
eTable 5. Prevalence of Chronic Conditions at Baseline Study Cohort from Zip Codes With Racial/Ethnic Diversity

eTable 6. Concordance Rate and EMS Transport Distance to ED by Race/Ethnicity
eTable 7. Comparison of Average Distance Between First and Second Most frequent EMS Destination Among non-Hispanic White Patients
eTable 8. Concordance in Destination ED Between Whites and non-Whites, by Age, Sex and Medicaid Eligibility
eFigure. Reference Emergency Departments in Boston City Zip Codes
eReferences.

This supplementary material has been provided by the authors to give readers additional information about their work.

© 2019 Hanchate AD et al. *JAMA Network Open*

eAppendix. Differences in Emergency Department Destination of Emergency Medical Services Transport of Co-located Patients by Race/Ethnicity and Geography

1. Sampling Universe

Eligible enrollees: Our study sample was drawn from the universe of Medicare enrollees during 2006-2012 (N=45.6 million in 2006 to 53.6 million in 2012); a sample was drawn from the universe for each year separately. For this study we selected those aged 66 and older and have continuous Fee for Service (FFS) coverage; for enrollees from 2006 to 2010, we required continuous coverage for 3 years, for enrollees from 2011 we required continuous coverage for 2 years and for enrollees from 2012 we required continuous coverage for 1 year. eTable 1 gives the counts for the selected subgroups for 2006 and 2012, overall and by race/ethnicity.

Zip codes with race/ethnic diversity: Using the residence zip code (reported in the Medicare beneficiary files) of continuously FFS enrolled members for each year, we stratified all zip codes by racial/ethnic diversity; a zip code was categorized as diverse if it was the residence zip code of >10 white, >10 black and >10 Hispanic eligible enrollees. Appendix Table 2 gives the composition of zip codes by racial/ethnic diversity for the 2009 study-eligible sampling universe; these figures were similar for the other years. An additional requirement of the study zip codes was that there be at least 5 EMS transports (to ED) for each of the three race/ethnic groups (from each zip code) during the study period.

The above table identifies qualifying 3,953 zip codes in 2009 data; similar analysis for all years identified a total of 4,175 unique zip codes, which formed the eligible zip codes for this study.

2. Sample Size

From the universe of eligible Medicare enrollees from the selected zip codes with race/ethnic diversity we obtained stratified random samples separately for each year. Medicare utilization records were only obtained for the sample cohorts. Following the rolling cohort design of national surveys (AHRQ's Medicare Expenditure Panel Survey and CMS' Medicare Current Beneficiary Survey), we stratified the sample for each year into three cohorts with each cohort followed for 1 to 3 years.[1-3] eTable 3 identifies the counts and follow-up periods for the 9 distinct cohorts in the study. Cohort 1 consisted of 123,791 enrollees sampled from the eligible universe in 2006; we obtained utilization records for 1 year (i.e., 1 year of follow-up). Cohorts 2 and 3 were also obtained from the 2006 universe but with longer follow-up periods. Cohort 4 is introduced in 2007, based on the 2007 universe, and replaces the retired Cohort 1. Cohorts 3 to 7 have 3 follow-up years. Cohort 8 is followed for 2 years and Cohort 9 for 1 year. Enrollees may be re-sampled in a subsequent cohort if they are no longer in an actively followed cohort. While the total sample count is 1,048,960, after excluding those re-sampled in multiple rounds, the number of unique enrollees was 864,750; we have reported this figure as the underlying sample size for all the ED visits examined in this study. For more about re-sampling, particularly from Massachusetts, see section 3 below.

3. Sampling by co-location

The desired sample count from the universe of each year was obtained by random sampling of enrollees stratified by zip code and race/ethnicity. Following were the sampling criteria.

1) Our preference was to obtain equal number of the 3 race/ethnic groups from each zip code; however, zip codes with racial/ethnic diversity varied considerably in the number of enrollees that could be sampled. The number that can be sampled is given by the number of the smallest race/ethnic group in the

zip code; for instance, if a zip code has 100 white enrollees, 20 black enrollees and 12 Hispanic enrollees, then we can sample at most 12 members of each group from the zip code. For better representation of the overall population we chose larger sample sizes from zip codes with larger number of enrollees that could be sampled.

2) The study data was designed as part of a larger study aimed at comparing ED use changes in Massachusetts vs. the remaining states in the country. Therefore, we over-sampled Massachusetts enrollees; approximately one-third of the total sample was from Massachusetts by design. In particular, for many zip codes, we were more likely to select all the black or Hispanic enrollees from the zip codes; consequently, these enrollees were likely to be re-enrolled in a later cohort.

eTable 4 provides the sampling rates by zip code categories: those with >100 enrollees from each race/ethnic group (category 1); those with 26 to 100 enrollees from each race/ethnic group (category 2) and those with 11 to 25 enrollees from each race/ethnic group (category 3). It presents the sampling numbers in Massachusetts and rest of the country separately.

4. Sampling weights

Our sampling strategy amounts to stratification of all eligible Medicare enrollees in these zip codes at two levels, first, by zip code, and second, by race/ethnicity. That is, all eligible enrollees in each zip code are stratified into four groups by race/ethnicity: Hispanics, (non-Hispanic) blacks, (non-Hispanic) whites and Others (all the remaining groups combined). As sampling of enrollees was done randomly from each group, the sampling probability for each selected enrollee is given by the ratio between the sample size and the total number of eligible enrollees from the racial/ethnic group in the zip code. The sampling weight is the inverse of this ratio.

Application of sampling weights leads to estimates generalizable to approximately 7 million underlying study-eligible Medicare enrollee population each year. This cohort is detailed in eTable 2 for 2009; there were 6,934,344 enrollees in the study-eligible zip codes.

5. Estimation

We estimated linear probability models of the following form:

$$\Delta y_{iz} = \beta_0 + \beta_1 * \Delta race_{iz} + \cdots + \alpha_1 * \Delta x_{1iz} + \cdots + t_i$$

where y_{iz} is a dichotomous indicator of transportation to the reference ED (and, in secondary analysis, to a safety-net ED) and Δy_{iz} denotes the difference between individual y_{iz} from the mean value at the zip code level. x_{iz} is an indicator of of patient characteristics (age, sex, dual Medicaid coverage, principal diagnosis cohort, comorbidity indicators). $race_{iz}$ is an indicator of race/ethnicity and $t_i$denotes dichotomous (fixed effects) indicators of each year of patient transport. We used least squares estimation and obtained standard errors robust to clustering at zip code level.[4]

eTable 1. Racial/Ethnic Composition of Eligible Medicare Enrollees

Year	Medicare enrollee subgroup		Race/ethnic group, %			
		Total # enrollees	White enrollees	Black enrollees	Hispanic enrollees	Other enrollees
2006	All Medicare enrollees	45,618,323	78.92	9.78	7.56	3.74
	# Medicare enrollees 66+ continuously enrolled in FFS during same year and following 2 years	20,180,062	86.19	6.68	4.16	2.97
2012	All Medicare enrollees	53,597,183	76.59	10.16	8.55	4.69
	# Medicare enrollees 66+ continuously enrolled in FFS during same year	24,658,341	83.63	7.45	5.04	3.88

Abbreviation: Fee For Service, FFS

© 2019 Hanchate AD et al. *JAMA Network Open*

eTable 2. Composition of Eligible Enrollees from Zip Codes With Racial/Ethnic Diversity, 2009

Zip code category	# zip codes	All enrollees	White enrollees	Black enrollees	Hispanic enrollees
All unique Medicare enrollees aged 66+ with continuous FFS coverage	38,423	20,249,187	17,221,032	1,410,898	930,727
Subgroup of enrollees in zip codes with racial/ethnic diversity (categories 1 to 3)	5,606	9,562,563	7,676,317	791,592	624,285
Subgroup of enrollees in zip codes with racial/ethnic diversity & 5 or more EMS trips in study data	3,953	6,934,344	5,484,597	616,343	505,735

Abbreviations: Fee For Service, FFS; Emergency Medical Services, EMS

© 2019 Hanchate AD et al. *JAMA Network Open*

eTable 3. Sample Size by Year and Follow-up Cohort Composition (Zip Codes With Diversity)

<table>
<tr><th rowspan="2">Cohort</th><th colspan="7">Year</th><th rowspan="2">All</th></tr>
<tr><th>2006</th><th>2007</th><th>2008</th><th>2009</th><th>2010</th><th>2011</th><th>2012</th></tr>
<tr><td>1</td><td>123,791</td><td>0</td><td>0</td><td>0</td><td>0</td><td>0</td><td>0</td><td>123,791</td></tr>
<tr><td>2</td><td colspan="2">132,127</td><td>0</td><td>0</td><td>0</td><td>0</td><td>0</td><td>132,127</td></tr>
<tr><td>3</td><td colspan="3">109,347</td><td>0</td><td>0</td><td>0</td><td>0</td><td>109,347</td></tr>
<tr><td>4</td><td>0</td><td colspan="3">108,432</td><td>0</td><td>0</td><td>0</td><td>108,432</td></tr>
<tr><td>5</td><td>0</td><td>0</td><td colspan="3">130,264</td><td>0</td><td>0</td><td>130,264</td></tr>
<tr><td>6</td><td>0</td><td>0</td><td>0</td><td colspan="3">107,050</td><td>0</td><td>107,050</td></tr>
<tr><td>7</td><td>0</td><td>0</td><td>0</td><td>0</td><td colspan="3">112,378</td><td>112,378</td></tr>
<tr><td>8</td><td>0</td><td>0</td><td>0</td><td>0</td><td>0</td><td colspan="2">119,481</td><td>119,481</td></tr>
<tr><td>9</td><td>0</td><td>0</td><td>0</td><td>0</td><td>0</td><td>0</td><td>106,090</td><td>106,090</td></tr>
<tr><td>Total enrollees each year</td><td>365,265</td><td>349,906</td><td>348,043</td><td>345,746</td><td>349,692</td><td>338,909</td><td>337,949</td><td>1,048,960</td></tr>
</table>

© 2019 Hanchate AD et al. *JAMA Network Open*

eTable 4. Sampling Design

Zip code category		Number of enrollees sampled from each zip code			
		White enrollees	Black enrollees	Hispanic enrollees	Others
All states except Massachusetts					
1. Zip codes with >100 enrollees of each of the 3 race/ethnic groups		14 to 22	14 to 22	14 to 22	1 to 2
2. Zip codes with 26 to 100 enrollees of each of the 3 race/ethnic groups		6 to 10	6 to 10	6 to 10	1
3. Zip code with 11 to 25 enrollees of each of the 3 race/ethnic groups		3 to 6	3 to 6	3 to 6	1
Massachusetts					
1. Zip codes with >100 enrollees of each of the 3 race/ethnic groups		1000 to 1500	60 to 100	60 to 100	30 to 50
2. Zip codes with 26 to 100 enrollees of each of the 3 race/ethnic groups		414 to 690	28 to 46	28 to 46	14 to 23
3. Zip code with 11 to 25 enrollees of each of the 3 race/ethnic groups		153 to 255	10 to 17	10 to 17	5 to 9

eTable 5. Prevalence of Chronic Conditions at Baseline Study Cohort from Zip Codes With Racial/Ethnic Diversity[a,b]
[For each condition, the No. (%) are reported below]

Covariate: Chronic Condition Comorbidity	All	Whites, non-Hispanic	Blacks, non-Hispanic	Hispanics
Alzheimer's disease, dementia	187,947 (40.4%)	84,418 (39.5%)	63,160 (46.7%)	35,446 (36.3%)
Anemia	278,507 (53.4%)	128,351 (58.2%)	90,404 (66.0%)	51,681 (62.1%)
Asthma	61,381 (11.4%)	25,257 (10.5%)	20,243 (14.8%)	13,919 (16.8%)
Atrial fibrillation	95,150 (23.7%)	57,286 (25.7%)	20,702 (14.3%)	14,595 (16.1%)
Cancer, breast	20,531 (4.6%)	11,701 (4.9%)	5,343 (3.7%)	2,986 (3.2%)
Cancer, colorectal	13,317 (2.8%)	74,77 (2.9%)	3,695 (2.6%)	1,821 (1.9%)
Cancer, endometrial	2,431 (0.54%)	1,398 (0.58%)	528 (0.34%)	444 (0.47%)
Cancer, lung	7,853 (1.7%)	4,559 (1.8%)	2,251 (1.7%)	836 (0.9%)
Cancer, prostate	20,655 (4.5%)	9,227 (4.4%)	7,774 (5.9%)	3,176 (3.4%)
Heart failure	231,891 (48.5%)	107,124 (47.5%)	75,667 (55.3%)	42,816 (50.9%)
Chronic kidney disease	195,705 (37.7%)	83,076 (35.2%)	71,362 (51.8%)	35,536 (42.7%)
COPD	148,393 (32.4%)	74,464 (32.8%)	44,111 (32.4%)	26,048 (30.8%)
Depression	162,556 (34.7%)	83,724 (35.6%)	42,732 (27.7%)	31,982 (37.2%)
Diabetes	230,939 (43.9%)	88,070 (39.5%)	83,671 (61.6%)	51,963 (63.1%)
Hip / pelvic fracture	22,928 (6.6%)	14,673 (7.4%)	3,568 (2.4%)	3,932 (4.4%)
Hyperlipidemia	274,448 (61.9%)	132,344 (62.1%)	79,709 (58.9%)	53,906 (63.2%)
Benign prostatic hyperplasia	48,132 (10.9%)	24,210 (11.0%)	13,410 (9.7%)	8,904 (11.0%)
Hypertension	414,680 (89.7%)	193,983 (88.8%)	129,706 (94.7%)	78,421 (91.4%)
Acquired hypothyroidism	98,198 (26.1%)	54,561 (28.1%)	21,951 (15.1%)	19,040 (23.2%)
Ischemic heart disease	298,409 (65.7%)	141,361 (65.3%)	91,512 (67.1%)	57,048 (63.7%)
Rheumatoid arthritis / osteoarthritis	225,336 (52.1%)	108,650 (52.3%)	67,921 (51.9%)	42,717 (47.3%)
Stroke / transient ischemic attack	85,754 (18.9%)	34,526 (18.1%)	32,283 (24.0%)	16,478 (19.3%)

Notes:
a) The prevalence rates are based on adjustment for stratified sampling.
b) The counts for All column includes the Other race/ethnic group for which we have not reported prevalence rates.

© 2019 Hanchate AD et al. *JAMA Network Open*

eTable 6. Concordance Rate and EMS Transport Distance to ED by Race/Ethnicity[a,b]

Patient subgroup	White patients, non-Hispanic		Black patients, non-Hispanic		Hispanic patients	
	Estimate	95% CI	Estimate	95% CI	Estimate	95% CI
Proportion of EMS-transported ED visits to the most frequent ED destination among white patients						
All zip codes	61.3%	[61.0%, 61.7%]	56.0%	[55.4%, 56.6%]	58.9%	[58.2%, 59.5%]
Subgroups of zip codes by # hospitals EDs in 10-mile vicinity						
0 to 1	72.9%	[72.3%, 73.6%]	72.3%	[71.3%, 73.4%]	70.3%	[69.0%, 71.6%]
2 to 4	62.4%	[61.8%, 63.0%]	57.2%	[56.1%, 58.2%]	59.5%	[58.3%, 60.6%]
5 or more	51.0%	[50.4%, 51.6%]	42.5%	[41.6%, 43.4%]	48.1%	[43.9%, 49.4%]
Largest cities						
Zip code in largest 16 cities	46.1%	[45.0%, 47.1%]	36.8%	[35.5%, 38.0%]	43.4%	[41.9%, 44.9%]
Average distance of EMS-transport to destination ED (mileage)						
All zip codes	6.53	[5.08, 7.98]	5.90	[4.80, 7.00]	5.90	[4.60, 7.49]
Subgroups of zip codes by # hospitals EDs in 10-mile vicinity						
0 to 1	7.20	[7.10, 7.30]	6.81	[6.64, 6.98]	6.91	[6.66, 7.15]
2 to 4	7.90	[4.04, 11.8]	6.51	[3.77, 9.25]	6.51	[3.77, 9.25]
5 or more	4.47	[4.41, 4.51]	4.28	[4.19, 4.38]	4.29	[4.21, 4.38]
Largest cities						
Zip code in largest 16 cities	4.28	[4.20, 4.37]	4.11	[4.19, 4.36]	4.11	[4.02, 4.22]

Abbreviations: Emergency Department, ED; Emergency Medical Services, EMS

Note:
a) All zip codes refer to the 217 zip codes that satisfy the inclusion criteria
b) Reported outcomes (proportion and distance) are adjusted for age, sex, primary diagnosis, comorbidities, Medicaid coverage, year and zip code location.

© 2019 Hanchate AD et al. *JAMA Network Open*

eTable 7. Comparison of Average Distance Between First and Second Most frequent EMS Destination Among non-Hispanic White Patients[a,b]

Patient subgroup	Average distance to ED (miles)		
	First most frequent destination ED ("reference ED")	Second most frequent destination ED	Difference [95% confidence interval]
All zip codes	4.8	10.8	6.03 [4.65, 7.40]
Subgroups of zip codes by # hospitals EDs in 10-mile vicinity			
0 to 1	5.8	10.4	4.55 [4.42, 4.67]
2 to 4	4.8	15.8	11.01 [7.68, 14.34]
5 or more	3.5	4.6	1.07 [1.0, 1.11]
Urban location			
Zip code in largest 16 cities	3.3	4.2	0.89 [0.83, 0.95]

Abbreviations: Emergency Department, ED; Emergency Medical Services, EMS

Note:
a) Difference in distance is defined as the distance to the second most frequent destination ED minus the distance to the first most frequent ED.
b) All patients from each zip code, regardless of race/ethnicity, were included in this estimation. Estimates of difference in mileage are based on a linear regression of mileage on an indicator of whether the destination ED was the first or second most frequent ED. No other covariates were included as our focus was on estimating the distance to ED of all patients located in different parts of the zip code, regardless of their clinical or sociodemographic characteristics.

© 2019 Hanchate AD et al. *JAMA Network Open*

eTable 8. Concordance in Destination ED Between Whites and non-Whites, by Age, Sex and Medicaid Eligibility[a]

Patient subgroup	Whites, non-Hispanic		Blacks, non-Hispanic		Hispanics	
	Estimate	95% CI	% difference in concordance rate between black and white patients	95% CI	% difference in concordance rate between Hispanic and white patients	95% CI
Age						
66-74	58.6%	[57.9%, 59.2%]	-4.2%	[-5.3%, -3.1%]	-2.0%	[-3.3%, -0.6%]
75-84	61.9%	[61.4%, 62.4%]	-4.5%	[-5.5%, -3.3%]	-1.6%	[-2.8%, -0.4%]
85+	62.3%	[61.7%, 62.9%]	-5.1%	[-6.4%, -3.7%]	-1.7%	[-3.2%, -0.1%]
Sex						
Men	60.0%	[59.5%, 60.6%]	-3.7%	[-4.9%, -2.6%]	-0.6%	[-1.9%, 0.8%]
Women	62.0%	[61.5%, 62.4%]	-6.0%	[-6.8%, -5.1%]	-3.7%	[-4.6%, -2.8%]
Medicaid eligibility						
Eligible	60.5%	[59.9%, 61.9%]	-6.6%	[-7.6%, -5.6%]	-4.2%	[-5.2%, -3.2%]
Not eligible	62.6%	[62.2%, 63.0%]	-5.7%	[-6.6%, -4.7%]	-2.0%	[-3.1%, -0.9%]
EMS Transport Type						
ALS	62.1%	[61.6%, 62.5%]	-5.0%	[-5.9%, -4.1%]	-2.8%	[-3.7%, -1.8%]
BLS	60.0%	[59.4%, 60.5%]	-5.6%	[-6.7%, -4.5%]	-2.0%	[-3.3%, -0.8%]
If destination teaching hospital ED						
Yes	56.1%	[55.7%, 56.5%]	-6.6%	[-7.4%, -5.8%]	-4.6%	[-3.0%, -1.2%]
No	66.3%	[65.9%, 66.7%]	-1.3%	[-2.1%, -0.4%]	-1.5%	[-2.4%, -0.5%]

Abbreviations: Emergency Department, ED; Emergency Medical Services, EMS; Advanced Life Support, ALS; Basic Life Support, BLS

Notes:
a) Concordance rate ratios were adjusted for age, sex, primary diagnosis, comorbidities, Medicaid coverage, year and zip code location.

eFigure. Reference Emergency Departments in Boston City Zip Codes (N=24,613 EMS Transports for non-Hispanic white patients)[a]

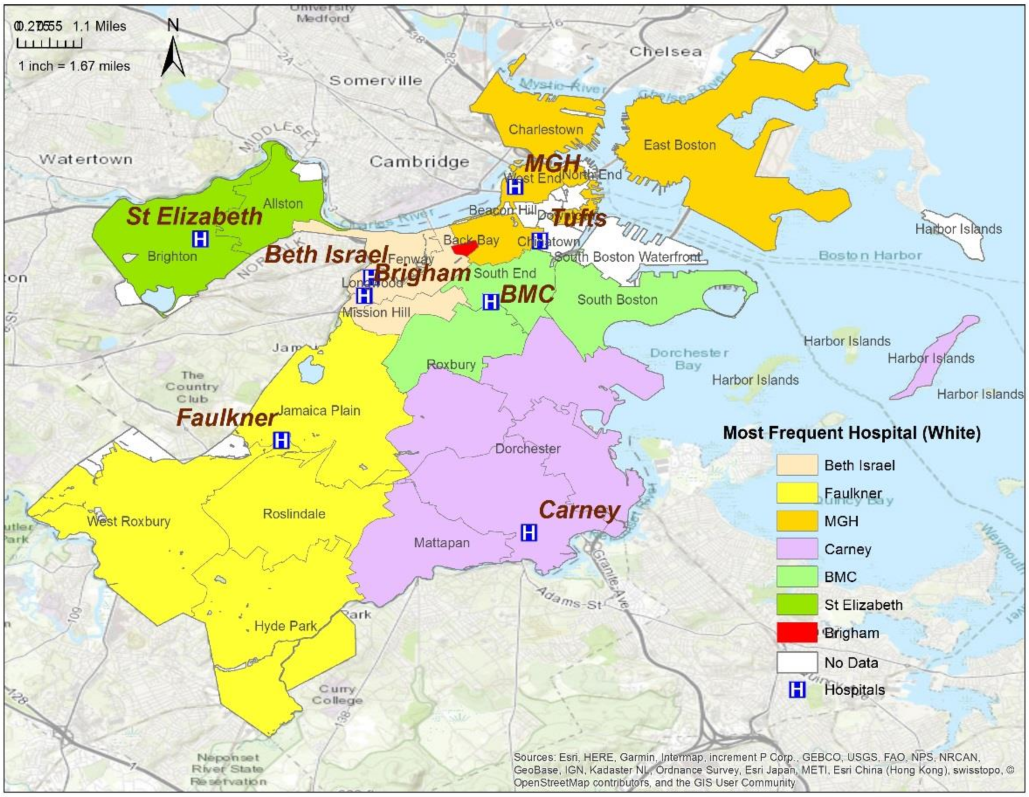

Abbreviations: Emergency Department, ED; Emergency Medical Services, EMS

Note:
a) Using all EMS transports in Boston city zip codes, we identified the most frequent (modal) ED destination for non-Hispanic white patients in each zip code. These are the "reference EDs". There are 8 EDs (hospitals) in Boston city. The map indicates the reference ED for each zip code.

eReferences

1. Agency for Healthcare Research and Quality. Medical Expenditure Panel Survey2015, Bethesda, MA.
2. Centers for Medicare & Medicaid Services. *Medicare Current Beneficiary Survey (MCBS).* https://www.cms.gov/Research-Statistics-Data-and-Systems/Research/MCBS/: Centers for Medicare & Medicaid Services;2016.
3. Korn EL, Graubard BI. *Analysis of health surveys.* Vol 323: John Wiley & Sons; 2011.
4. Hansen BE. *Econometrics.* https://www.ssc.wisc.edu/~bhansen/econometrics/2018.
